# Development and Characterisation of Biodegradable Polymeric Composites Enhanced with Nanoparticles for Antimicrobial and Biomedical Applications

**DOI:** 10.3390/polym18010051

**Published:** 2025-12-24

**Authors:** Aaruci Agarwalla, Waleed Ahmed, Tif AlMeqbaali, Fatma Alsayegh, Mehraj ud din, Lateifa Abdulla Butti, Akela Ghazawi, Ali H. Al-Marzouqi, Mushtaq Ahmad Khan

**Affiliations:** 1 Chemical and Petroleum Engineering Department, College of Engineering, United Arab Emirates University, Al Ain P.O. Box 15551, United Arab Emirates; 2 Engineering Requirements Unit, College of Engineering, United Arab Emirates University, Al Ain P.O. Box 15551, United Arab Emirates; 3 Mechanical and Aerospace Engineering Department, College of Engineering, United Arab Emirates University, Al Ain P.O. Box 15551, United Arab Emirates; 4 Critical Care Division, Integrated Hospital Care Institute, Cleveland Clinic Abu Dhabi, Al Ain P.O. Box 15551, United Arab Emirates; 5 Department of Medical Microbiology and Immunology, College of Medicine and Health Sciences, United Arab Emirates University, Al Ain P.O. Box 15551, United Arab Emirates; 6 Zayed Center for Health Sciences, United Arab Emirates University, Al Ain P.O. Box 15551, United Arab Emirates

**Keywords:** metal polymeric composites, antimicrobial efficacy, injection moulding

## Abstract

In this study, metallic nanoparticle polymeric PLA composites such as Silver (Ag)+PLA, Nickel (Ni)+PLA, Copper (Cu)+PLA, and Copper Oxide (CuO)+PLA were developed using injection moulding to study the antimicrobial efficacy of the developed polymeric composites against several Gram-positive and Gram-negative pathogenic strains like *Enterococcus faecalis*, *Staphylococcus aureus*, *Klebsiella pneumoniae*, *Salmonella Poona*, *Pseudomonas aeruginosa*, and *Escherichia coli.* SEM and EDS were used to identify the surface morphology and elemental composition of Ni+PLA, Ag+PLA, Cu+PLA, and CuO+PLA composites, which showed specific metal dispersion and surface roughness, which have effects on antimicrobial activity. Thermal characteristics analysed by TGA and DSC showed an increase in thermal stability and crystallisation properties because of nanoparticle integration. All four composites demonstrated high efficacy against the tested bacterial strains, achieving an overall reduction of approximately 70% in all samples. A more substantial decrease of 98% was observed across all strains in each composite, except for a slightly lower efficacy of 97% in the Ni+PLA and Ag+PLA composites against *Enterococcus faecalis* and *Pseudomonas aeruginosa*. The potential for such antimicrobial materials and their characteristics make the current investigation particularly innovative for the medical field, and such antimicrobial materials would be extremely beneficial to reduce HAIs and drug-resistant organisms that otherwise complicate patient care and lower the efficiency and quality of health care provided.

## 1. Introduction

Hospital-acquired infections (HAIs) or nosocomial infections refer to those types of infections that patients develop during their hospitalisation as a result of medical treatment of another condition. These infections develop within 48 h or more of admission to hospitals and were neither incubating nor clinically apparent infections at the time of admission. Some of the most widespread causes of HAI include *Enterococcus faecalis*, *Staphylococcus aureus*, *Klebsiella pneumoniae*, *Salmonella Poona*, *Pseudomonas aeruginosa*, and *Escherichia coli* strains. These pathogens are notoriously resistant to antibiotics [1,2]. Their continual presence on clinical surfaces further advances the chances of patient complications and adds to growing healthcare expenses. Thus, HAIs represent a critical public health issue that demands ongoing attention and an action plan to mitigate their impact on patients and health care systems worldwide [2,3]. The increasing prevalence of multidrug-resistant organisms has decreased the efficiency of the conventional methods of disinfection; thus, there is a pressing need to find materials that can resolve microbial persistence at the point of contact [4].

Nosocomial and community-acquired infections are primarily spread by touching infected surfaces [5]. Medical devices and surfaces in hospitals (e.g., indwelling catheters, surgical instruments, and door handles) are highly susceptible to contamination by pathogens [6,7]. The exponential increase in new antibiotic drugs in the post-antibiotic era cannot keep up with the rise in bacterial resistance, posing a severe threat to human health. As a result, antimicrobial materials have been used as viable alternatives to eliminate or limit microbial growth, thereby preventing nosocomial and biofilm-related diseases. These materials address the critical needs of public health systems. The creation of a highly resistant microenvironment against antibiotics and disinfectants by biofilms in particular rationalises the need to incorporate antimicrobial characteristics into the substrates of medical devices, instead of merely depending on external sterilisation processes [5,8,9].

Antimicrobial materials, by virtue of their design, prevent microbial transmission and biofilm-associated disease on their surfaces or surrounding environments by killing or inhibiting microbial growth with biocidal coatings, surface-bound antimicrobials, or pathogen-repellent surfaces [5,10]. These materials are engineered using a variety of substances, including metals, polymers, ceramics, and composites. Common examples include silver, copper, zinc, antimicrobial peptides, carbon-based nanomaterials, and bioactive antimicrobial nanoparticles, which have been incorporated into polymeric matrices to minimise microbial contamination and enhance infection prevention [11,12,13,14]. The modification of polymeric material with antimicrobial nanoparticles has become one of the most promising solutions, because of the capacity of nanoparticles to impart prolonged antimicrobial protection, enhanced material stability, and compatibility with conventional manufacturing methodology [14,15,16,17,18,19].

Copper and copper oxide have the best broad-spectrum antimicrobial properties and are generally long-lasting against bacteria, viruses, and fungi, and thus good biomedical surface candidates [14,18]. Nickel nanoparticles (Ni-NPs) serve as antimicrobial agents for biomedical and industrial applications since they exhibit substantial antimicrobial effects against a variety of bacterial and fungal pathogens [20]. Silver nanoparticles (AgNPs) exhibit antimicrobial activity against various bacteria, fungi, and viruses [21]. Though the AgNPs are efficient against both Gram-positive and Gram-negative bacteria, including their drug-resistant strains, the antimicrobial efficiency of AgNPs can be enhanced when combined with antibiotics or other antimicrobial agents. Collectively, these nanoparticles made of metals demonstrate the wide range of antimicrobial approaches that are being investigated to improve biomedical devices [21].

New developments in sustainable biomaterials have put into the limelight the importance of bio-derived and environmentally friendly antimicrobial systems in biomedical applications. Plant-based or green nanoparticle synthesis is an approach that has received particular momentum as an alternative to chemically synthesised nanomaterials. As an example, MCF-7 breast cancer cells exhibited selective anticancer activity against gold nanoparticles produced by Thymus vulgaris synthesised using porous silica, demonstrating the potential application of phytochemicals in the synthesis of nanoparticles [22]. Similarly, the Centella asiatica-coated silver nanoparticles embedded in electrospun nanofiber dressing demonstrate high levels of antimicrobial activity and wound-healing relevance [23]. In line with these tendencies, recent examination of the protein corona has emphasised that the surfaces of nanoparticles promptly obtain biological identity in vivo, which has a great impact on cellular interactions, biodistribution, and therapeutic performance [23,24]. Even though the nanoparticles employed in the current work were obtained commercially, contextualising our injection-moulded PLA composites within this larger framework highlights their applicability to new, sustainable, nano-enabled antimicrobial materials [22,23,24]. The methodology presented here can be expanded in the future to adhere to green-synthesised nanoparticles and corona-informed material design. Despite the fact that the current study is concerned with antimicrobial performance, the complete biomedicalization of such nanocomposites has been investigated in the literature [24].

Another manufacturing process relevant to the medical device industry comes from injection moulding with antimicrobial materials or antimicrobial composite manufacturing. Using antimicrobial materials in composites makes medical devices more antimicrobial in that they prevent hospital-acquired infections (HAIs). For plastics injection moulding, for instance, antimicrobial materials are effective in the manufacturing of parts designed to prevent the propagation of bacteria, viruses, and fungi [10,25]. Moreover, antimicrobial materials can be composites within injection moulding resins, so that the medical devices created maintain antimicrobial qualities throughout their life [10].

Numerous studies have been conducted on 3D printing and its application in developing antimicrobial materials for biomedical usage. In contrast, very few highlight the use of injection moulding in the development of antimicrobial medical devices or biomedical applications (utilising antimicrobial agents) [16]. Injection moulding can play a crucial role for products with high-volume production needs by increasing the production speed in comparison to 3D printing, and parts or products produced by 3D printing may not have the same surface finish or mechanical properties as those made through injection moulding, which can be critical in medical applications where sterility and strength are essential [16,26]. Although many studies have presented PLA–nanoparticle composites, most studies have utilised 3D printing or surface coating, and few have focused on injection moulding, which is a scalable fabrication technique and industrially feasible to manufacture medical equipment. In addition, past studies were usually concentrated on individual nanoparticles; here, four (Ag, Cu, CuO, Ni) are being tested under the same conditions, compared with clinically relevant Gram-positive and Gram-negative pathogens, giving a direct comparative analysis of the performance of nanoparticles [27]. This paper gives a direct comparison of various metal-based PLA composites that have been fabricated with injection moulding, and the approach fills a major void in scalable antimicrobial material. Assessment of these composites under the same fabrication and biological testing conditions enables the study to go a long way in determining how various nanoparticle chemistries impact antibacterial activities, thermal behaviour, and material morphology [28,29,30,31].

We have examined in this study the antimicrobial performance of Silver (Ag), Nickel (Ni), Copper (Cu), and Copper Oxide nanoparticles (CuO) within polymeric composites. The proposed study will compare the antimicrobial activity of the developed composites in order to identify which formulation offers the most efficacy against a wide range of Gram-positive and Gram-negative bacteria. It also discusses how they may be implemented in a health care setting, in response to the urgent requirement of new antimicrobial approaches to deal with the increasing challenge of multidrug-resistant pathogens with the goal of improving the morbidity and mortality of patients, with direct impact on the length of stay and overall financial burden to the community at large. The paper also goes to assess their potential in being incorporated into health care applications where long-lasting antimicrobial performance is critical.

## 2. Material Properties

Copper nanoparticles: Due to their extraordinary physical, chemical, and electrical characteristics, copper nanoparticles are highly beneficial in every sphere of electronics and materials, to catalysts. The complex surface area, electrical and thermal conductivity, and optical effects, such as surface plasmon resonance, result in copper nanoparticles that render similar properties to bulk copper but at a more useful scale. For instance, copper is known to have excellent antimicrobial properties; thus, copper nanoparticles can be applied in systems where infection control is needed, like medical textiles/coatings or the textiles themselves. Furthermore, copper nanoparticles are very catalytic in reactions; their small average particle size provides them with more active sites and reactivity than bulk copper. They can be used for many applications, since copper oxidises and forms copper oxides [32]. Furthermore, since they possess high antimicrobial properties and good infection control, they can be incorporated into biocompatible scaffolds like cellulose and chitosan to reduce excessive nanoparticle agglomeration/leaching and consistent antimicrobial activity and controlled release for multiple usages. Copper can be classified as a low magnetic material, especially when associated with others, which is interesting because bulk copper is not magnetic; this is good for magnetic storage devices. They act as very catalytic agents, with high reaction efficiency via rapid velocity in reactions such as methanol synthesis and hydrogenation [33]. In addition, they maintain malleability and ductility, which is useful within composites and surface coatings [34].

These copper nanoparticles are also investigated for targeted drug delivery and cancer treatment for better drug stabilisation and precision [35], while their biocompatibility and antimicrobial characteristics make them useful in wound dressings and tissue engineering for effective healing of wounds and reduced infection. In the field of electronics, the nanoparticles have distinguished electrical conductivity, so they are used in conductive coatings and inks, while also being a precursor for many other electronic components [36].

Copper oxide nanoparticles: Copper oxide nanoparticles (CuO NPs) are highly multifunctional nanomaterials that find extensive use in electronics, medical applications, and environmental studies, as a result of their physical properties, size, shape, surface area, and density [37]. Copper oxide nanoparticles are acid-soluble, insoluble in aqueous solutions, and stable in most air conditions. They have oxidation and reduction potential, and their increased surface area creates more active sites for reactivity, which is beneficial in catalytic, antimicrobial, and environmental studies. CuO NPs are advantageous from an optical standpoint for photocatalytic and solar applications due to their direct band gap from 1.2 to 2.1 eV, which is helpful when tuning for plasmonic properties for sensing applications and optoelectronic applications [38]. Furthermore, NPs have antiferromagnetic properties that can be controlled at nanoscale levels for information storage and magnetically guided drug delivery. Hardness, strength, thermal stability, and antimicrobial properties of composites are greatly increased when CuO NPs are incorporated; they are nanoscale materials that have great potential across diverse fields and many applications in scientific research and industrial applications [39].

In addition, CuO NPs are extensively used across a variety of types of materials and are frequently used on ceramics, glass, catalysts, superconductors, thermoelectric materials, and sensors. For example, they are found in magnetic storage devices, near-infrared absorbers/filters, semiconductors, photothermal uses, gas sensors, and photoconductors [40]. In addition, they are heavily used in solar energy conversion and catalysis combustion catalysts for rocket fuels, which utilise CuO NPs and NPs to enhance the burn rate for homogeneous propellants and reduce pressure to facilitate universal combustion [38,40]. In medicine, copper oxide nanoparticles (CuO NPs) are antimicrobial agents, drug delivery systems, wound healing materials, and biosensors; they are coated onto fabrics and medical equipment for antimicrobial properties, enhanced durability, and biodistribution during delivery, and are used in wound healing and biomolecule sensing applications for glucose and cholesterol biosensing [41].

CuO NPs were recently incorporated into smart bandages that change colour when infected, providing a form of aesthetics and diagnosis without the need to travel to the doctor’s office. Furthermore, the research is increasingly being applied to CuO NPs in implantable sensors that assess chronic disease (diabetes) from inside the body. Such operations and potential involve copper oxide nanoparticles outside of typical medical use [41].

Silver nanoparticles: Silver nanoparticles (AgNPs) have a range of properties that make them a nanoparticle of interest for use in environmental applications, electronics, and medicine. These properties include antimicrobial solid-state activity, unique optical properties, enhanced electrical conductivity, high surface area, biocompatibility, thermal stability, and chemical stability [42]. For example, Ag NPs are effective in medical instrument/textile coating and water disinfection as antimicrobial agents. In addition, AgNPs have potential for sensors, imaging, and photothermal applications due to their optical properties, specifically SPR. Moreover, in electronics and flexible devices, Ag NPs are effective because they enhance the conductivity of composites in devices [43]. Ag NPs also have the potential to facilitate excellent catalytic activity for many chemical reactions, as the surface area to volume ratio creates more reactivity, increasing the reactions facilitated by access. In addition, Ag NPs can be thermally stable, meaning that at high temperatures, the properties are still maintained. In addition, properties can be maintained at room temperature without change or oxidation degradation. The reactivity/stability of the Ag NPs also depends significantly on their size and shape [44].

Biocidal and disinfectant properties of silver nanoparticles are recognised as antibacterial agents, and further investigation is currently underway with respect to AIDS treatment [44,45]. The efficacy of pathogen resistance of E. coli and Staphylococcus aureus is established with the addition of small quantities (~0.1%) of silver nanopowder in various inorganic matrices, with similar results across pH and oxidative conditions. Moreover, Ag NPs have been incorporated into drug delivery systems as they are noncytotoxic, provide minor toxicity for biocompatibility, and are extensively used for biological investigations in genetic diagnostics. Yet aside from medical intervention, silver nanoparticles (Ag NPs) possess commercial viability in consumer goods. Expected enhanced longevity and anticorrosion with antibiosis qualities encourage the use of such nanoparticles in washers and dryers, refrigerators, children’s toys, food containers, and even construction materials. Silver nanoparticles are also being studied as better wound-dressing materials with controlled release profiles, in the hope that silver can improve the healing of chronic wounds and minimise scarring [45].

In addition, AgNPs are also undergoing trials in advanced medical coatings on hospital equipment and surfaces, which would offer them permanent safeguarding against multidrug-resistant bacterial infections. These developments indicate that silver nanoparticles are being incorporated into modern medicine [46].

Nickel nanoparticles: The distinctive properties of nickel nanoparticles (NiNPs), such as excellent catalytic activity in hydrogenation and oxidation reactions, combined with a high surface area-to-volume ratio that enhances efficiency, make them extremely useful [47]. In magnetic data storage and biomedical imaging applications, NiNPs are highly valuable because of their ferromagnetic behaviour. In addition, due to their ability to retain good electrical conductivity, they are suitable for use in electronics, such as conductive inks and sensors [48]. Nickel nanoparticles (NiNPs), owing to their plasmonic properties, are utilised in photothermal therapy and sensing applications. In addition, they are potential hydrogen storage technologies because of the capability to store hydrogen gas [49]. Typical applications of NiNPs include catalysis, lubricants, fuels, conductive pastes and coatings, electrodes, capacitors, ceramic and sintering additives, and more [47,49].

Moreover, NiNPs can be oxidised by air or moisture, though they are stable in different conditions. NiNPs can be engineered for biocompatibility, making them suitable for biomedical applications such as drug delivery and imaging [48,49]. NiNPs can be designed to be biocompatible, allowing them to be used in biomedical applications like drug delivery and imaging to deliver drugs to a particular tissue or cell [48,49]. NiNPs can serve as effective carriers in drug delivery systems and can be used in biomedical research and practice, such as drug delivery and imaging. Another important role of NiNPs is that they can make various imaging methods have better contrast [49]. Most recent studies are exploring the use of nickel nanoparticles to reduce post-surgical infections, via the use of these nanoparticles in innovative implant coatings that can release antimicrobial agents in response to bacterial presence, preventing the disease.

Also, NiNPs are being explored as a potential for non-invasive imaging in early oncology detection, using the particularities of NiNPs’ magnetic properties to enhance the accuracy of diagnosis. The continued benefits that have been realised emphasise the increased value of nickel nanoparticles in novel health care applications [49]. Table 1 below describes the properties of different nanoparticles used to develop the composites for injection moulding [32,34,37,38,41,42,46,47].

## 3. Method

### 3.1. Injection Moulding

**Figure 1:**
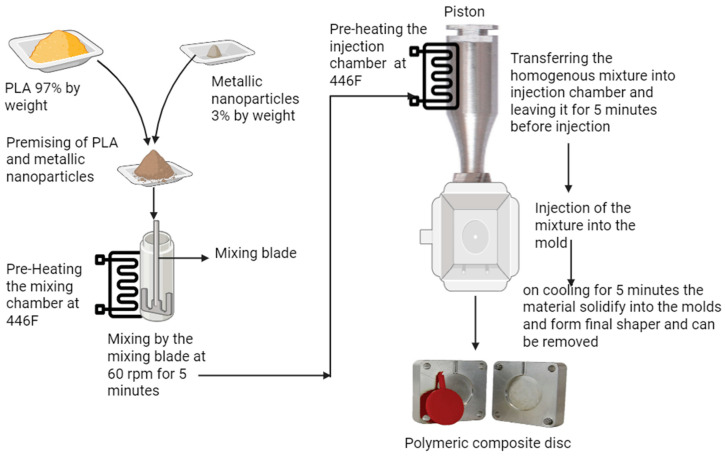
Graphical summary of the injection moulding process.

To fabricate metal nanoparticle PLA polymeric composite discs (Ag+PLA, Cu+PLA, Ni+PLA, CuO+PLA), the metal nanoparticles and the PLA were mixed in the ratio of 3% metal nanoparticles by weight and 97% polylactic acid (PLA) by weight. All metal nanoparticles (Ag, Cu, Ni, CuO) used in this study were sourced and utilised without any further modification from Nanografi (Ankara, Turkey) [50]. This specific ratio, 3%, was intentionally selected to align with previous studies on copper–PLA composites produced via 3D printing, which exhibited excellent antimicrobial properties, ensuring consistency and comparability in the material’s performance [31,51,52,53,54]. The process began with the precise weighing of the PLA and respective metal nanoparticles (Ag, Cu, Ni, or CuO) before mixing. Figure 1 illustrates the stages of the process. At the time of weighing, the metal nanoparticles were added to the PLA base material to facilitate prepositioning for the subsequent injection moulding step for the composite polymer, with adequate dispersion and amount and proper heating. Subsequently, the following materials were added to the high shear mixing unit to obtain a heterogenous composite mixture. This is relevant to the field of study because it means that when composite development occurs, there will be proper inclusion of the metal nanoparticles within the PLA. The Benchtop Model B-100 injection moulder runs at a temperature below 230 °C to ensure that the PLA is melted/viscous enough for the composite polymer to run consistently into the moulds. This is relevant to the field of study because it means that sufficient injections from a single batch can be carried out to allow for composite development to align with mechanical testing and antimicrobial testing for final composite development. Mould preheating eliminates defects like warping or cold spots in the fabricated composite discs, which may jeopardise the integrity and quality of final specimens. The resulting polymeric composite discs were used for antimicrobial testing.

Furthermore, this study aims to develop metal–PLA hybrids that are cost-effective and easily translatable to industrialisation on a mass scale. This will facilitate standardised evaluations and enable performance evaluations of all polymeric composites under the same parameters, as all hybrid metallic nanoparticle composites will be created with the same weight percent of metal nanoparticles and PLA (Ag+PLA, Cu+PLA, Ni+PLA, CuO+PLA).

Composite materials were prepared with an intentional approach to promote uniformity and the intended use of the resultant composites. For instance, for the melting of the PLA and the PLA/metal nanoparticle homogeneity, the injection moulding machine mixing chambers were filled with materials and mixed at 60 rpm for 5 min. This is a good enough rotational speed to promote even distribution, but not so much to clump and separate/inappropriately segregate nanoparticles or create uneven distributions of nanoparticles.

Once the mixing process was completed, the mixture was then injected into the moulding chamber, which was maintained at a temperature of 446 °F (230 °C), the same as that of the mixing chamber. The material was then left in the moulding chamber for 5 min, allowing it to reach thermal equilibrium and ensuring that the entire mixture had fully melted and maintained its uniformity before being injected into the mould. Here, circular steel moulds with a diameter of 40 mm (explicitly chosen to meet the size requirements for antimicrobial testing) were used for the development of the composites, but before the injection of the samples into the mould, the moulds were preheated, either by placing them in an oven at 80 °C for 5 min or heated them with a heat gun to facilitate the smooth flow of the molten composite during injection. Preheating the moulds before the injection of the samples is important to ensure the smooth flow of the molten composite during injection. Mould preheating also reduces defects such as warping or cold spots on the fabricated composite discs that could threaten the integrity and quality of final specimens. After cooling and solidification, specimens were removed from the moulds for subsequent antimicrobial testing. Therefore, temperature-controlled moulding guaranteed that testing standards would be consistently and reliably applied to all specimens.

### 3.2. Morphological Characterisation (Methods)

SEM was employed to observe the surface morphology of the composites under investigation (Ni+PLA, Ag+PLA, Cu+PLA and CuO+PLA) for particle distribution and polymer–filler interfacial characteristics. The presence of metal or metal oxides in the PLA matrix (homogeneously distributed) was evaluated by analysing the elemental distributions via EDS, working with the help of SEM. Metal nanoparticle composites were subjected to SEM and EDS using a JEOL JSM-6-10PLUS/LA scanning electron microscope and an energy dispersive spectrometer sourced from Tokyo, Japan. Prior to assessment, the sample was sputter-coated with a very thin layer of gold particles to enhance conductivity and improve imaging capabilities. All imaging was performed at 500X.

### 3.3. Thermal Characterisation (Methods)

TGA was performed under a nitrogen atmosphere to assess thermal stability and degradation of the composites in question and provide the researcher with a greater understanding of where each filler may have contributed to the departure of the polymer and remnants. DSC was conducted to study such processes as Tg and cold crystallisation and melting in order to obtain the crystallinity and the effect the metal and oxide constituents may impose on the nucleation.

DSC was performed using DSC equipment from TA Instruments with the help of Trios software (version 4.4.1.41651). Thermogravimetric analysis was conducted using a TA Instruments TGA Q500, a high-performance thermogravimetric analysis instrument, which is specially designed with a sensitive thermobalance and low-mass furnace to measure changes in weight with high precision, both sourced from New Castle, DE, USA. To evaluate the thermal stability and degradation behaviour of the metal nanoparticle PLA composites, the analysis was carried out in the temperature range of 25–800 °C.

### 3.4. Methodology: Antimicrobial Testing-Time Kill Assay

A nanoparticle–polymer hybrid composite disc (Copper–PLA, Silver–PLA, Nickel–PLA, and Copper Oxide–PLA), 3% by nanoparticle weight, was prepared using the injection moulding method. A polyethene sheet (commercially available) was used as a control. Polyethene (PE) was chosen as the control sheet when testing antimicrobials because it is chemically inert, non-degradable, and has no inherent antimicrobial activity at all. This is in line with ISO 22196:2011 [55], the international standard of evaluating antibacterial activity on plastics, where it is defined that a polyethene-based neutral film should be used as a reference substrate to cover and compare the microbial inoculum [55,56].

In comparison, polylactic acid (PLA) is susceptible to hydrolytic breakdown in humid and hot environments, leading to acidic end groups and the modification of surface energy that allows for the adhesion and viability of microorganisms. Since PLA is not chemically stable throughout the incubation period, it cannot be used as a reliable negative control [57,58,59,60,61]. In this case, therefore, PE was taken as the standardised baseline material with which antimicrobial benchmarking would be conducted [57,58,59,60,61].

The hybrid composites were grounded or sanded for 3 min using sandpaper with a 200-grit strength to ensure proper exposure of the surface nanoparticles to the bacteria. The antimicrobial efficacy of the prepared hybrid composites was tested against clinically relevant organisms that are responsible for most of the HAIs. Standard ATCC strains of *Escherichia coli* (25922), *Staphylococcus aureus* (25923), *Klebsiella pneumoniae*, *Acinetobacter* spp., *Pseudomonas aeruginosa*, and *Enterobacter* spp. were used to contaminate the composite surfaces for testing.

**Figure 2:**
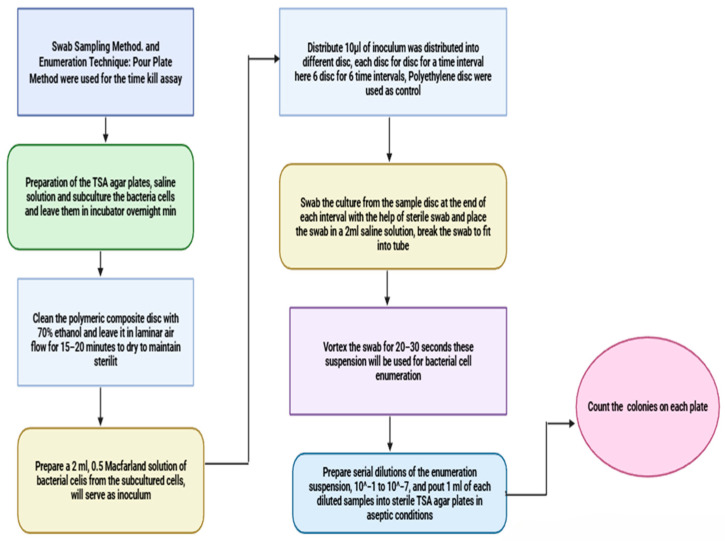
Flowchart showing the methodology of the time kill assay.

Protocol: The Swab Sampling Method (CMMEF Chapter 3) [62] and Enumeration Technique (Pour Plate Method, APHA 9215 B, BAM Jan 2001, Chapter 3) [63] were used for the time kill assay. To measure the bacteria’s antimicrobial activity, the agar plate diffusion technique, as outlined in protocol CCFRA 1.1.4.2003, was used [64]. The entire setup and set of procedures were developed by our team, following microbiology norms and ISO 22196 [55]. For the antimicrobial properties, samples of bacteria with certified standards were tested and subsequently stored in the lab as a quality control measure. Figure 2 depicts a flowchart of the methodology of the time kill assay.

Although the antimicrobial behaviour of the Ni-PLA, Cu-PLA, Ag-PLA, and CuO-PLA composites has been substantively illustrated in this study, their applicability in biomedical research also varies on biocompatibility.

To confirm the sterility of the experimental materials, PLA and nanoparticle–PLA hybrid composite discs were surface-sterilised with 70% ethanol and air-dried for 10 min under aseptic conditions. The surfaces of the sterilised discs were then swabbed using sterile cotton swabs, and the swabs were streaked on TSA plates and incubated at 37 °C for 24 h. Absence of growth on these plates confirmed the sterility of the discs.

To prepare the inoculum, overnight TSA cultures were inoculated with isolated colonies and resuspended in 2 mL of saline solution (0.85%). The suspension was then adjusted to a turbidity corresponding to a 0.5 McFarland standard with the help of a densitometer. A 10 µL aliquot of this standardised suspension, which is equivalent to 10^6^ CFU/mL, was pipetted onto each of seven PLA discs (control group) and seven nanoparticle–PLA hybrid composite discs (test group). The discs were left in a biosafety cabinet at room temperature to avoid contamination, and read at 0, 10, 20, 30, 40, 50, and 60 min. In addition, a 2 h exposure and 24 h exposure were also read for an extended bactericidal effect. At each reading, recovery of the bacteria on the discs was attempted using a sterile cotton swab. For those discs that dehydrated the inoculum, recovery was attempted with a cotton swab hydrated with saline first. Each cotton swab was placed into a 2 mL snap mL cap tube containing saline and vortexed for 20–30 s to ensure any recovered bacteria that might be stuck to the cotton were dislodged. The solution extract was subsequently serially diluted 10-fold with saline, and viable dilutions were transferred in triplicate onto TSA plates. Colony-forming units (CFUs) were counted, TSA plates were incubated at 37 °C, and the viable bacteria that remained per disc were determined.

A time-dependent reduction in bacterial viability was observed at all time points investigated. However, at 60 min there were no colonies present on the nanoparticle–PLA hybrid discs. Results from the 2 and 24 h exposure and incubation time validated these results and demonstrated a 100% antibacterial effectiveness with only 60 min exposure.

#### Bacterial Count Collection

Serial dilution and colony count at each time point were used to determine the number of adhered bacteria on PLA and nanoparticle–PLA composite discs. All swab suspensions were vortexed for 20–30 s, then diluted ten-fold (10^−1^ to 10^−7^ to 10^−8^) in sterile saline solution, and 100 µL of each dilution was plated in triplicate on TSA plates and incubated at 37 °C for 24 h. Only plates showing 30–300 CFUs were counted for the viable count. In the case of greater than 1000 CFUs, more dilute dilutions (10^−1^) were excluded. For example, if approximately 4.5 × 10^3^ CFUs were noted on the discs, the 10^−2^ dilution was appropriate for determining viable count since it yielded 45 separate colonies. More dilute dilutions beginning at 10^−3^ were excluded, as they did not yield numbers greater than 20 to count. Viable count was calculated based on CFUs counted at each respective time point compared to the control (PLA) group. This approach allowed for the determination of time-dependent bactericidal efficacy of the nanoparticle–PLA composites. An identical experiment was performed with additional viability assays at extended time points (2 h and 24 h) to confirm extended antimicrobial activity. The testing method complied with CCFRA 1.1.4:2003 regulations, and the testing method and microbiological study were defined in accordance with ISO 22196:2011 and CCFRA 1.1.4:2003 [55,64]. The values from all three replicates were averaged and reported as mean CFU/mL Findings were processed through Minitab (Release 21.3.1).

## 4. Results

### 4.1. Morphological Characterisation (Results)

(i) **SEM:**

**Figure 3:**
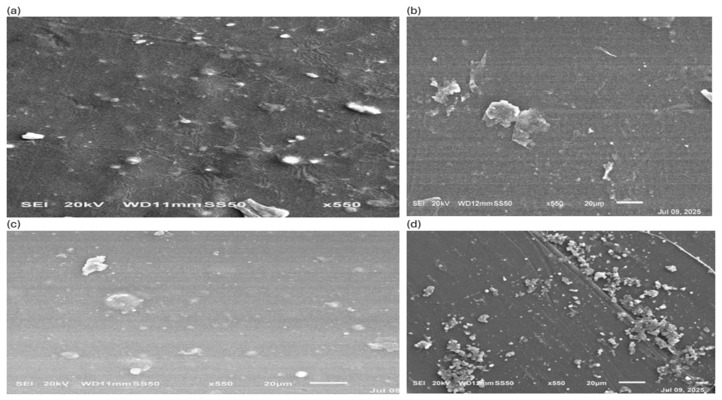
(**a**) SEM image of Ni+PLA; (**b**) SEM image of Ag+PLA; (**c**) SEM image of Cu+PLA; (**d**) SEM image of CuO+PLA.

The surface morphology differences from one metal to another in the PLA can be observed in the SEM micrographs of Ni+PLA, Ag+PLA, Cu+PLA, and CuO+PLA composites (Figure 3a–d). The Ni+PLA compound (Figure 3a) exhibited a smooth surface with a few bright particulates scattered all over it, indicating the limited exposure of nickel on the surface and possible sub-surface fixation. In the Ag+PLA composite (Figure 3b), a comparatively smooth matrix with a few flaky and irregularly distributed deposits was observed, which suggests that the localised aggregations of silver had partial surface adhesion. The Cu+PLA composite (Figure 3c) showed scattered tiny protrusions and irregular deposits, which indicated moderate agglomeration of the particles on the PLA surface. Contrastingly, the CuO+PLA composite (Figure 3d) had an extremely rough surface where the clusters were tightly packed, indicating that more deposition occurred on the surface and there was stronger incorporation of the smaller CuO particles than metallic Cu.

(ii) **EDS:**

**Figure 4:**
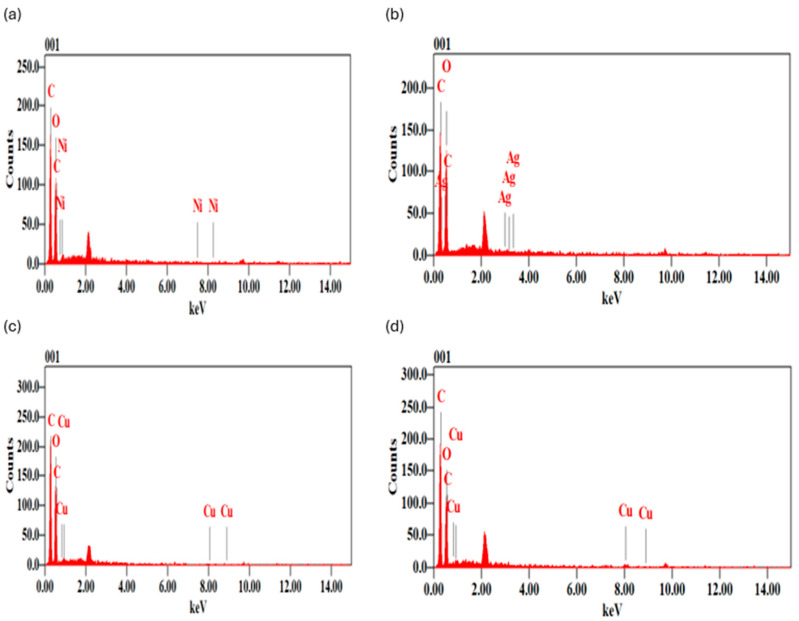
(**a**) EDS of Ni+PLA; (**b**) EDS of Ag+PLA; (**c**) EDS of Cu+PLA; and (**d**) EDS of CuO+PLA.

From Figure 4a–d, we observe that the EDS spectra confirmed the elemental composition of the PLA metal composites with distinctly strong peaks at the positions of the elements carbon (C K(a) at 0.28 keV) and oxygen (O K(a) at 0.52 keV), present in the backbone of the PLA. Besides these matrix peaks, there are other weak but distinct signals that were observed to be of the incorporated metals. In the case of Ni+PLA, a Ni kα peak was detected with a weighted contribution of 0.83 mass (0.19 atom). Ag Lα in Ag+PL A was hardly visible, at 0.05 mass% (0.01 atom%). The Cu Kα signal of the Cu+PLA composite was weak, indicating 0.11 mass (0.02 atom) of Cu. Conversely, the CuO-PLA sample had the strongest Cu Kα peak, with 1.97 mass% (0.42 atom) content. Table 2 provides a summary of the quantitative EDS data. EDS analysis of the quantitative data is shown in Table 2 below.

### 4.2. Thermal Characterisation (Results)

(i) **TGA:**

**Figure 5:**
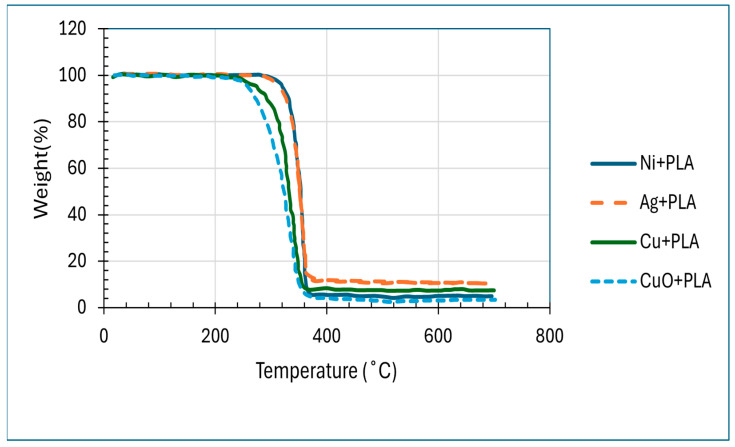
TGA results for Cu-PLA, Ag-PLA, Ni-PLA, and CuO-PLA.

**Figure 6:**
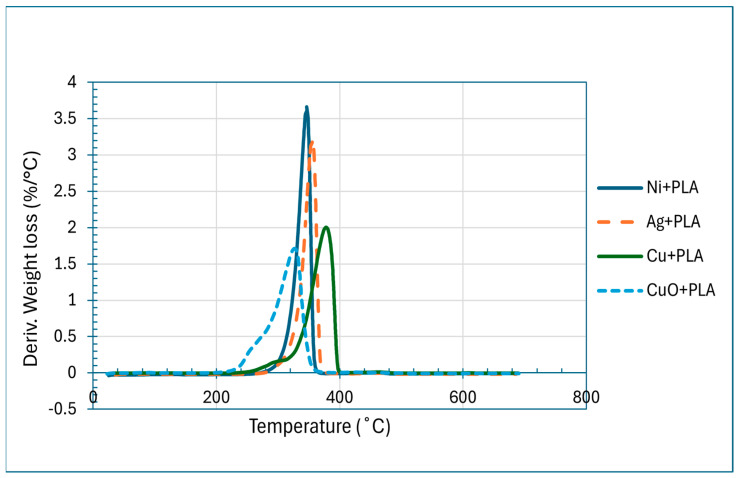
DTG results for Ni-PLA, Ag-PLA, Cu-PLA, CuO-PLA.

Thermogravimetric analysis (TGA) was used to determine the thermal stability of PLA nanocomposites that were reinforced using Cu, Ag, Ni, and CuO nanoparticles. According to the TGA curves (Figure 5), it is observed that all the composites degraded above 355–440 °C, and this is much higher than the degradation temperature of pure PLA (320–330 °C). Specifically, Cu-PLA (390 °C), 92.5% Ag-PLA (370 °C), 90.5% Ni-PLA (355 °C) 95%, and CuO-PLA (380–390 °C) 94% showed a weight loss. The weight loss rate was slower at temperatures above 390 °C, with further degradation, which was considered to be small, due to the metallic interfaces: 2.5% at 430 °C with Cu, 2 percent at 440 °C with Ag, 2.5% at 400 °C with Ni, and 3% at 410 °C with CuO. The remainder was ascribed to the presence of the inorganic filler content. The total mass-loss behaviour of the composites is thus explained in Figure 5, and the related DTG curves in Figure 6 demonstrate the rate of degradation and the temperatures at which the composites decompose the most.

(ii) **DSC:**

**Figure 7:**
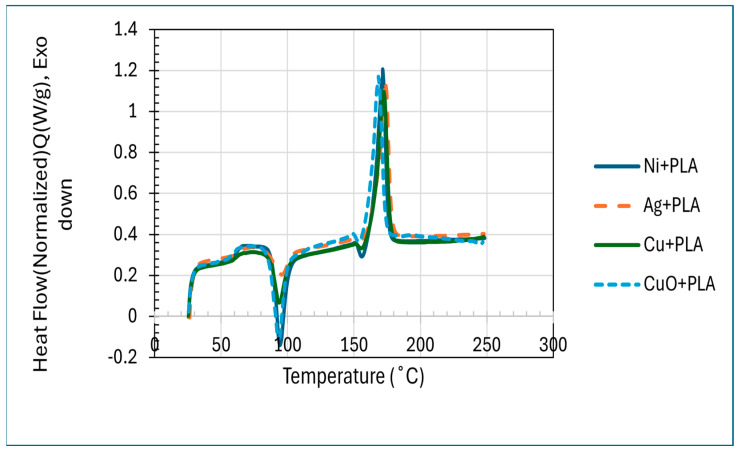
DSC thermogram of Ni+PLA, Ag+PLA, Cu+PLA and CuO+PLA.

As shown in Figure 7, the DSC thermograms of Ni+PLA, Ag+PLA, Cu+PLA, and CuO+PLA composites have a glass transition temperature (Tg) near 59.9 °C, 60.4 °C, 60.6 °C, and 58.4 °C, respectively, and none of the additives significantly changed chain segmental mobility in the amorphous phase. Ni+PLA (ΔHcc = 22.5 J g^−1^), Ag+PLA (ΔHcc = 8.8 J g^−1^), Cu+PLA (ΔHcc = 12.4 J g^−1^), and CuO+PLA (ΔHcc = 22.5 J g^−1^) all exhibited cold-crystallisation peaks of approximately 93.6 °C, 95.0 °C, 93.4 °C, and 93.0 °C, respectively, suggesting that all composites were largely amorphous after the processing, but different in the degree of crystallisation during heating. The following melting transitions were obtained: Ni+PLA: 170.9 °C (ΔHm = 35.7 J g^−1^), Ag+PLA: 172.6 °C (ΔHm = 41.0 J g ^−1^), Cu+PLA: 172.2 °C (ΔHm = 37.5 J g^−1^), and CuO+PLA: 168.3 °C (ΔHm = 37.8 J g^−1^). The values of the calculated crystal percentage, 100% crystalline PLA = 93 J g^−1^, were approximately 14, 35, 27, and 16% with Ni+PLA, Ag+PLA, Cu+PLA, and CuO+PLA, respectively. 

### 4.3. Antimicrobial Testing (Results)

**Figure 8:**
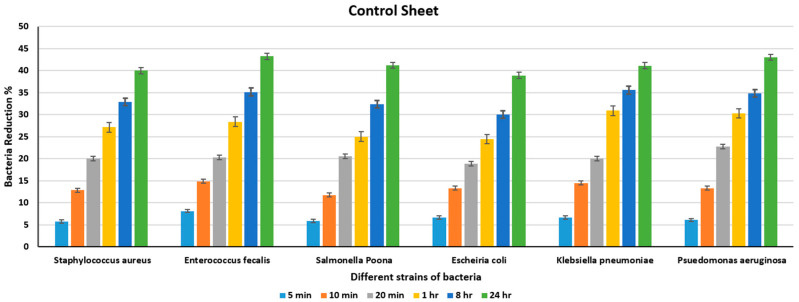
Illustrates the reduction in the total bacterial count percentage for the control sheet at various time intervals for different bacteria.

Four sets of antimicrobial polymeric composites, developed using a known composition with the aid of the injection moulding method, were labelled as samples 1, 2, 3, and 4. A commercially available polyethylene sheet was used as a control to evaluate the antimicrobial effectiveness of the developed metal–PLA composites. Antimicrobial efficiency was evaluated over different intervals of time, 5 min, 10 min, 20 min, 1 h, 8 h, and 24 h, against various strains of Gram-positive and Gram-negative bacteria *(Escherichia coli*, *Staphylococcus aureus*, *Pseudomonas aeruginosa*, *Klebsiella pneumoniae*, *Salmonella Poona*, *and Enterococcus faecalis)*. Table 3 and Figure 8 display the bacterial counts of different strains on the control sheet after various time intervals.

**Figure 9:**
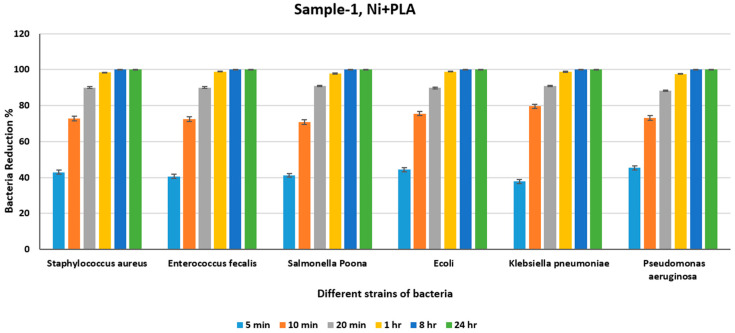
Shows the reduction in the total amount of bacterial percentage for the Ni+PLA sheet at different intervals of time for various bacteria.

Figure 8 and Table 3 show that there is no significant reduction in the number of bacteria, even after 8 h, particularly for *Staphylococcus aureus* and *Enterococcus faecalis*, where more than 40% of the bacteria remain present on the surface of the plastic control sheet after 24 h. The minimal number of bacteria in the control sheet after 24 h was found in *Enterococcus faecalis* and *Pseudomonas aeruginosa* (1880 and 2000, respectively), which are still significant numbers, demonstrating limited natural reduction, as the plastic surface acting as the control sheet has no antimicrobial efficacy of its own. Table 4 shows the count of different bacteria for the Ni+PLA sheet for various periods, and Figure 9 shows the percentage reduction in the bacterial count at the end of different intervals for the Ni+PLA sheet.

**Figure 10:**
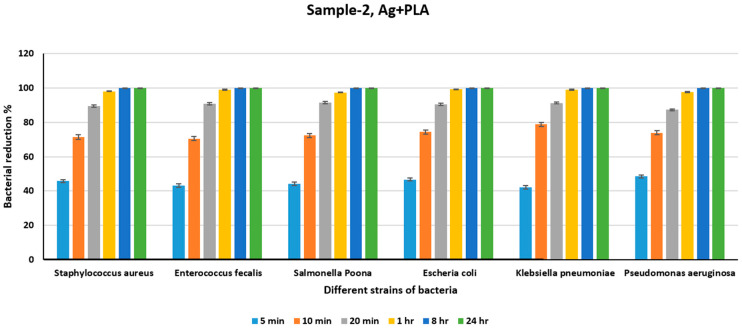
Shows the reduction in the total count of bacterial percentage for the Ag+PLA sheet at different intervals of time for different bacteria.

Table 4 shows the count of different bacteria for the Ni+PLA sheet for different periods, and Figure 9 shows the percentage reduction in the bacterial count at the end of different intervals for the Ni+PLA sheet. From Table 4 it can be deduced that more than 98 percent of the different strains of bacteria were killed at the end of 24 h and from Figure 9 it can be seen that Ni+PLA is effective in killing more than 70 percent of the different strains of all the bacterial in just 10 min and 98 percent of the different strains in just 1 h. We can see an almost 100% reduction in all the different strains of bacteria at the end of 24 h. Table 5 shows the count of different bacteria for the Ni PLA sheet for various periods, and Figure 10 shows the percentage reduction in the bacterial count at the end of different intervals for the Ag+PLA sheet.

**Figure 11:**
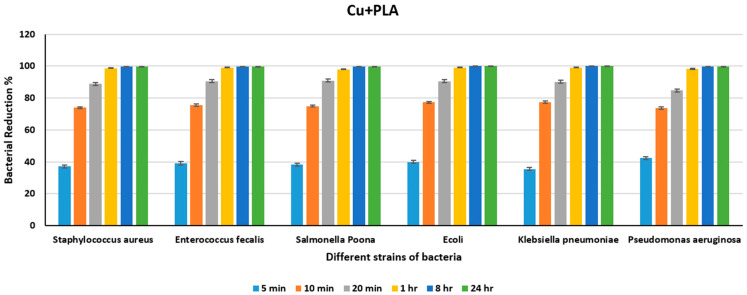
Shows the reduction in the total count of bacterial percentage for the Cu+PLA sheet at different intervals of time for different bacteria.

From Table 5, we can see that there is a substantial reduction in bacterial counts in just 20 min for all the different strains of bacteria upon exposure to Ag+PLA. From Figure 10, we can say that there is only an approximate 70% reduction in all the various strains of bacteria in 10 min, except in Klebsiella pneumoniae, which shows a 78% reduction, and an approximate 90% reduction in bacteria in 20 min in comparison to that of Ni+PLA. However, we can see near-total destruction of the bacteria, with a more than 98% reduction for all the different strains at the end of 1 h and 100% in 24 h. In Figure 5 we can also see a significant reduction in the bacterial count from 5 min to 10 min compared to 10 min to 30 min or between subsequent other intervals. Table 6 shows the count of different bacteria for the Cu+PLA sheet for different periods, and Figure 11 shows the percentage reduction in the bacterial count at the end of varying intervals for the Cu+PLA sheet.

**Figure 12:**
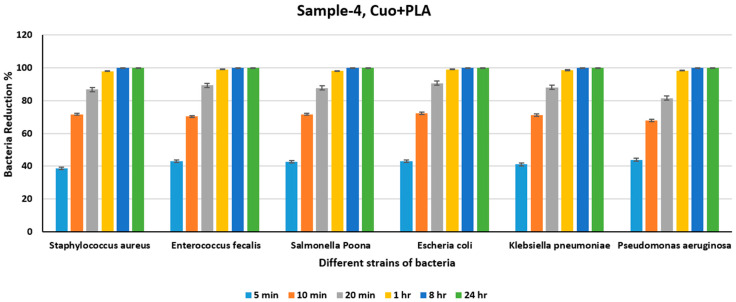
Illustrates the reduction in total bacterial count percentage for the CuO+PLA sheet at various time intervals for different bacteria.

From Table 6 and Figure 11, we observe a substantial reduction in bacterial counts within 20 min for all bacterial strains upon exposure to Cu+PLA. From Figure 11 we can see that there is only approximately 74% reduction in all the different strains of bacteria in 10 min, an approximate reduction of 90% of the bacteria in 20 min, and near-destruction of the bacteria, that is, more than 98% reduction, for all the different strains at the end of 1 h when exposed to Cu+PLA, showing the high antimicrobial efficacy of Cu ions in Cu+PLA composites. The antimicrobial properties of Cu nanoparticles are responsible for reducing the bacterial count due to the antimicrobial activities of the Cu ions, which lead to bacterial cell death. The release of Cu ions is fast initially and then decreases, leading to the prolonged antimicrobial efficacy of these metal nanoparticles over several days [65]. From Figure 11, we can see a significant reduction in the bacterial count from 5 min to 10 min, compared to the interval from 10 min to 1 h or between subsequent intervals. Table 7 shows the count of different bacteria for the CuO+PLA sheet for various time periods, and Figure 12 shows the percentage reduction in the bacterial count at the end of varying intervals for the CuO+PLA sheet.

From Table 7, it is evident that there is a substantial reduction in bacterial counts within 20 min for all bacterial strains upon exposure to CuO+PLA. From Figure 12, we can observe that there is an approximate 70% reduction in all strains of Bacillus within 10 min and an approximate 90% reduction in bacteria within 20 min compared to CuO+PLA. However, we can see that there is near-total destruction of the bacteria, resulting in more than a 98% reduction for all strains at the end of 1 h when exposed to CuO+PLA, demonstrating the high antimicrobial efficacy of CuO ions in CuO+PLA composites. Thus, like Ni+PLA and Ag+PLA, CuO+PLA shows a drastic reduction in viable cell counts within 20 min for all studied bacteria, as shown in Table 6 and Figure 12. The metal and metal oxide ions, Ni, Cu, Ag, and CuO of these composites justify the antibacterial effectiveness. These metal and metal oxide nanoparticles form in the composite and induce the release of metal ions and generation of ROS to act upon the cell membrane and the cellular machinery, disrupting the cellular machinery and the cell membrane to induce cell death [65]. For example, with their interactive antibacterial impact it becomes easier to achieve higher microbial counts as they suppress growth. Therefore, the results show that the composite materials Ni+PLA, Cu+PLA, Ag+PLA, and CuO+PLA have varied usefulness in biomedical, environmental, and industrial fields. The incorporation of metal particles as composite materials provides a strong means by which the antibacterial effectiveness and functionality of such materials in polymer applications can be increased.

Upon comparison of the antimicrobial activity of the four composites, it was found that there was a more than 98% reduction in all strains of bacteria, except for Ni+PLA and Cu+PLA, which were slightly less effective, resulting in a 97% reduction for Salmonella Poona and Pseudomonas Aeruginosa. This may be because silver is more potent in antimicrobial activity than copper and nickel [66]. However, there is a 99.9% reduction in bacteria for all samples after 8 h. This implies that there is a slight variation in effectiveness in the short term but uniform effectiveness with regard to long-term exposure on all materials. It may be due to the fact that various antimicrobial effects (membrane disruption, ROS, enzyme inhibition) eventually reach the same endpoint, or that bacterial repair mechanisms are not able to withstand the continued exposure.

Including metal particles in polymeric materials is a valuable strategy to enhance the antibacterial performance and utility of such materials. It should, however, be noted that although certain metals like nickel are effective antimicrobials, they have also been reported to have cytotoxic effects in biological systems. This cytotoxicity has potential risks in clinical use, particularly in cases where these materials come into contact with human tissues. Thus, thorough in vitro cytotoxicity assays and then in vivo biocompatibility research should evaluate the safety profile of these nanoparticle composites. This biological testing is the only way to appropriately establish a level of antimicrobial efficacy versus biocompatibility for safe in vivo use [66,67]. These safety issues can be determined by comprehensive toxicity studies that will inform the next steps to take these types of materials from the lab bench to the bedside. In addition, future studies can help better define compositions for the most effective antimicrobial efficacy with minimal adverse biological effects [68].

## 5. Discussion

### 5.1. Morphological Characterisation (Discussion)

(i) **SEM:** The morphological observations define the importance of metal type and chemical nature in the dispersion behaviour in PLA. The surfaces of Ni+PLA and Ag+PLA are smoother and contain fewer visible particulates, and this implies that they are weaker in interfacial compatibility with particles that exist at the bottom of the surface rather than uniformly on the surface. Cu+PLA had moderate dispersion, which showed partial compatibility and agglomeration on small scales. CuO+PLA exhibited a high level of clustering and surface coverage, indicating an increase in the affinity of copper oxide with the PLA matrix. The surface features are essential, as the more irregular and uneven morphologies are related to better bioreactivity and possible antimicrobial effectiveness. Likewise, it has been found that oxide nanoparticles have a greater surface contact with PLA than metallic ones, which increases surface roughness and operational performance [69,70].
(ii) **EDS:** The EDS analysis shows different degrees of metal presence in the composites, which are consistent with the morphologies on the surface. Ni+PLA displayed a low Ni Kα signal and 0.83 mass% Ni as an indication of the low surface sensitivity and retention of nickel in the PLA, which is comparable to the low particulates of SEM. Ag+PlA had the lowest incorporation rate (0.05 mass percentage) and had a trace Ag Lα peak, which further supports the SEM result of a predominantly smooth surface with few deposits. The incorporation of Cu+PLA was slightly higher (0.11 mass) with a faint Cu Kα signal, which confirms the presence of scattered protrusions found in SEM micrographs. Comparatively, CuO–PLA exhibited the strongest Cu Kα peak and highest incorporation (1.97 mass%), which is directly correlated to the highly roughened cluster-rich morphology observed in SEM. The results indicate that the oxide form of copper is not only easier to incorporate into PLA, but it is also more surface-active than its metallic equivalent. The same past research has already found that oxide nanoparticles exhibit more interfacial relationships with polymer matrices and also have a higher surface expression than elemental metals [67]. The findings of SEM and EDS correlate with these results. Combined, SEM and EDS offer complementary systems of metal incorporation and distribution in PLA composites. The Ni+PLA and Ag+PLA composites have smooth morphologies, thus leading to weak or trace EDS signals, whereas the Cu+PLA and the CuO+PLA have rougher surfaces and stronger metal peaks, respectively. Significantly, the high level of Cu Ka in CuO+PLA is a quantitative verification of the presence of dense surface agglomerates in SEM, and the fact that Ni and Ag are embedded in the sub-surface is why they are less visible on the surface. The combination of these two methods, therefore, gives a consistent image of the role of metal type and oxidation state on the structural integration of nanoparticles, as well as the surface accessibility of nanoparticles in PLA [69,70].

EDS was not eliminated, since its use is to detect the presence of nanoparticles, surface exposure, and dispersion, critical parameters which directly affect the antimicrobial properties of PLA when containing metallic nanoparticle composites, and XRD was not suitable to substitute EDS in this application [71]. As illustrated in Pušnik et al. [70], where nanoparticle-containing PLA composites were studied, XRD was not useful with a small amount of nanoparticles mixed into the PLA, with the authors specifically pointing out that with a large dispersion, XRD analyses are not productive, and instead, direct methods like SEM should be utilised to determine the presence and distribution of nanoparticles [70]. This, therefore, is in direct support of our plan that EDS is the valid and requisite method to ascertain the presence and distribution of metal nanoparticles, and as such, we humbly seek to keep the EDS results [70,71]

### 5.2. Thermal Characterisation (Discussion)

(i) **TGA:** From Figure 5 and Figure 6, we observed that the improvement in degradation temperature observed over pure PLA had been confirmed to be because of nanoparticle incorporation, which enhances thermal stability of the composites [72]. This is due to the barrier effect of nanoparticles, which blocks the diffusion of volatile degradation products, and high interfacial interactions, which limit chain mobility and raise the energy of activation to decompose [73]. Ni-PLA was by far the most stable composite with the highest retained mass over higher temperatures, implying that it has the best interfacial reinforcement, and the CuO-PLA also showed significant improvement, although with a broader degradation profile, indicating some catalytic effect. Cu-PLA was more resilient to temperature (390 °C), whereas Ag-PLA was better than pure PLA but had a lower degradation initiation than the others, presumably because of less strong polymer filler interactions. On balance, these results indicate that the addition of nanoparticles is an effective method to relocate PLA degradation to higher temperatures, and Ni-PLA and Cu-PLA can be used as the most thermally stable systems, which is why they are especially applicable in high-temperature conditions [72,73].
(ii) **DSC:** The DSC results in Figure 7 show that all the composites had glass transition temperatures near those of neat PLA of about 59.9 °C (Ni+PLA), 60.4 °C (Ag+PLA), 60.6 °C (Cu+PLA), and 58.4 °C (CuO+PLA), meaning that the additives did not have any significant influence on the mobility of the amorphous chain segments. Nevertheless, a clear difference was displayed in crystallisation and melting behaviour. Ni+PLA, with a cold-crystallisation peak of around 93.6 °C (ΔHcc = 22.5 Jg^−1^) and a melting enthalpy of 35.7 Cg^−1^, demonstrated the lowest crystallinity (approximately 14 per cent), indicating a reduced nucleation strength of nickel, although it had comparable thermal transitions to pure PLA. Ag+PLA, on the other hand, exhibited a cold-crystallisation peak of approximately 95.0 °C (ΔHcc = 8.8 J g^−1^) and a melting enthalpy of 41.0 J g^−1^, which then gave the highest crystallinity (~35%). The large difference between the melting enthalpy and cold crystallisation is evidence of the fact that silver greatly contributed to the nucleation and allowed the formation of stable crystalline structures before heating, which is in agreement with previous studies that the nucleation barrier is reduced by Ag nanoparticles and promotes the formation of stable crystalline domains [74,75]. To cold-crystallise, Cu+PLA showed an eluent peak at approximately 93.4 °C (ΔHcc = 12.4 J g^−1^) and a melting enthalpy of 37.5 J g^−1^, which is related to a moderate level of crystallinity (approximately 27 percent), and a large fraction of crystal domains were formed during heating, consistent with reports [70,75] that copper-based additives enhance PLA ordering to a lesser extent than silver. CuO+PLA, where the cold-crystallisation peak was of the order of 93.0 °C (−ΔHcc = 22.5 J g^−1^) and the melting enthalpy was 37.8 J g^−1^, attained a crystallinity level of about 16%, and this implies that most of its crystalline structure must have been generated upon heating, and the oxide phase was a weaker nucleating agent than the metallic equivalents, in accordance with previous studies demonstrating lower nucleation efficiency of the metal oxides. These results combined suggest that although the additives did not produce any significant effect on Tg, the effect on ΔHcc and ΔHm defined the degree and mode of crystallisation, with silver exhibiting the highest nucleation capacity, copper a moderate impact, and nickel and copper oxide minimal effect. Also, the difference in crystallinity relates to the significance of additive chemistry and phase in guiding the crystallisation pathways in polymers [74,75]. The enhanced performance of the metallic silver and copper over the nickel and copper oxide is an indication that the metallic fillers offer better nucleation sites owing to their surface energy properties and well-developed interfaces with the PLA matrix. This supports the fact that the crystallisation process cannot solely rely on thermal treatment but also on the physicochemical character of the filler, which determines the stability and packing of crystal nuclei developed in the polymer [75,76,77,78].

### 5.3. Antimicrobial Testing (Discussion)

Research into the antimicrobial activities of metallic nanoparticles and their oxides has garnered considerable attention in recent years. The antimicrobial activity of metallic nanoparticles with PLA against several Gram-positive and Gram-negative bacteria (*Escherichia coli*, *Staphylococcus aureus*, *Pseudomonas aeruginosa*, *Klebsiella pneumoniae*, *Salmonella Poona*, *and Enterococcus faecalis)* was studied over different periods. In the plastic control sheet, we observed a decline in the number of bacteria for all the different strains, but still a significant number was found at the end of 24 h. The cause of the decrease in bacterial counts on the plastic sheets can be ascribed to a combination of environmental factors, the microbial species, and the colonisation pattern, which result in a gradual decrease in bacterial populations [6,79]. The reason why polyethene (PE) was used as the control material is in line with the established literature in antibacterial surfaces testing, where PE or LDPE is used as the inert reference polymer. This method is comparable to ISO 22196 [55] and various articles that use PE as the neutral standard when assessing antimicrobial materials. As opposed to that, unmodified PLA is biodegradable and prone to hydrolysis during incubation, where it may change its surface chemistry and possibly affect microbial viability. Nevertheless, we acknowledge that including a pure PLA control would provide an internal comparator specific to the PLA matrix and allow for clearer isolation of nanoparticle-driven antimicrobial effects. This represents a limitation of the present study, and future work will incorporate both PE and nanoparticle-free PLA controls to enable fully resolved comparative analysis [80,81,82,83].

It was found that nickel nanoparticles with PLA (Ni+PLA) showed excellent antimicrobial efficacy, with approximately 98% reduction in bacterial count in just 1 h and approximately 100% in 24 h. The polymeric composite Ni+PLA, in which the Ni ions are present, is responsible for reducing the bacterial count over the period. The antimicrobial composite produces Ni ions, which possess strong antibacterial properties. These properties are responsible for disrupting the bacterial cell membrane and interacting with the cellular components of the bacteria, thereby inhibiting DNA replication, disrupting their metabolic processes, and ultimately leading to cell death [84]. In a similar study by Ahghari et al. [84], magnetic Nickel nanoparticles were synthesised, and their antimicrobial efficiency against *S. aureus* and *E. coli*, the Gram-positive and Gram-negative bacteria, respectively, was investigated. It was found that more than 80% of the bacteria were reduced in the presence of magnetic nickel nanoparticles after 18 h [84].

In the antimicrobial efficacy of Ag+PLA, a reduction of approximately 70% in bacteria was observed in just 10 min, except for *K. pneumoniae*, where a reduction of approximately 78% was observed. The reason for the susceptibility of *K. pneumoniae* may be that the Ag ions released from the PLA matrix preferentially target specific metabolic pathways in Klebsiella or exhibit potential synergies between bacterial surface proteins and silver nanoparticles unique to Klebsiella [85,86]. Ehlashkar et al. [87] developed an AgNPs@chitosan-NaF composite to study its antimicrobial susceptibility and efficacy in combating *K. pneumoniae* biofilms. It was found that *K. pneumoniae* was highly susceptible to AgNPs, and there was a synergistic effect of silver nanoparticles, chitosan, and sodium fluoride in combating *K. pneumoniae* biofilms [87]. It was further observed that there was a 98% decline in all the different strains in just 1 h, and a complete 100% decline in 24 h, similar to what was observed in Ni+PLA. The reason for the decline is that Ag ions released from Ag+PLA disrupt the cell membrane and interfere with the metabolic pathways of the bacteria, leading to the generation of ROS and ultimately cell death. In a similar study by Bayraktar et al. [88], Ag nanowire-loaded PLA composites with varying Ag compositions (1 wt.%, 2 wt.%, 3 wt.%, and 4 wt.%) were synthesised using 3D printing, and their morphological, thermal, and antimicrobial properties were studied. The antimicrobial properties were studied against Gram-positive and Gram-negative bacteria, *S. aureus* and *E. coli*. It was found that there was 100% antibacterial efficiency against *E. coli* in just 2 h, and the antibacterial efficacy continued for 24 h across all varying compositions. In contrast, for *S. aureus*, 100% bacterial efficacy was noted for a 4% Ag composite at 8 h [88]. It was further noted that there was a much greater reduction in bacterial number between 5 and 10 min than between 10 and 20 min or any subsequent interval, because of Ag+PLA. This was because the release of Ag ions is fast initially and then decreases, leading to the prolonged antimicrobial efficacy of these metal nanoparticles over several days [16,88].

Similar outcomes were observed for Cu+PLA to those of Ag+PLA, with nearly 98% reduction in 1 h and a complete elimination of about 100 percent for all the different strains in 24 h because of the release of copper ions from Cu+PLA, which were responsible for the death of the bacteria. In a study by Ahmed et al., the researchers developed copper–PLA composites (90% copper and 10% PLA) with the help of 3D printing, and it was found that the developed copper–PLA composites showed 99.5% antimicrobial efficacy against both Staphylococcus aureus and Escherichia coli in just 20 min [6,89]. In a similar study, Kudzin et al. [89] developed copper–PLA composites using the direct current (DC) magnetron sputtering method, which involved the surface modification of melt-blown poly(lactide) non-wovens with copper. This approach enabled the study of the physico-technical and biological characterisation of the developed composite material. It was found that the developed composite had antimicrobial efficacy against Gram-negative (*Escherichia coli*), Gram-positive (*Staphylococcus aureus*) bacteria, and antifungal efficacy against Chaetomium globosum, with potential application as an antimicrobial material [89].

Similar results to Ag+PLA and Cu+PLA were observed in CuO+PLA, with a 98% reduction in 1 h and 100% elimination in 24 h of all different strains. The antimicrobial properties of CuO nanoparticles are responsible for reducing the bacterial count due to the antimicrobial activities of the CuO ions, which lead to bacterial cell death [90]. Ren et al. [91] studied the development of copper oxide nanoparticles using thermal plasma technology and characterised the antimicrobial properties of the resulting materials. It was found that the developed copper oxide nanoparticles showed high antimicrobial efficacy against methicillin-resistant *Staphylococcus aureus* (MRSA) and *Escherichia coli* [91].

The antimicrobial efficacy of Ni+PLA, Ag+PLA, Cu+PLA, and CuO+PLA from the agar plate disc diffusion shows that all the developed composites showed an excellent antimicrobial effectiveness and a very similar pattern, that is, approximately 90% reduction in 20 min and nearly 100 percent reduction in 8 h. Pusnik et al. [70] produced PLA films using various amounts (0–3 wt.%) of Ag, ZnO, and TiO2 nanoparticles in the melt extrusion and film development steps, and the films were examined for improvements to their mechanical characteristics, as well as their ability to combat bacteria such as E. coli and S. aureus. The modified nanofilms successfully battled E. coli and showed weak antimicrobial activity against S. aureus; after 6 h, the Ag-nanoparticle-modified films were the most effective against both microbes. According to published studies and the results obtained from this study, injection moulding can be used to produce antimicrobial PLA-based composites that can effectively remove both types of bacteria. Although it has been demonstrated that metal nanoparticles, such as Cu, CuO, Ag, and Ni, when incorporated into PLA composites, exhibit significant antimicrobial properties, the safety aspects of their use in humans should always be carefully considered. Research studies have shown that these nanoparticles can be biocompatible under controlled concentrations, although concerns regarding cytotoxicity, systemic accumulation, and chronic exposure effects remain. Therefore, critical in vitro and in vivo screening are needed to support their clinical safety and regulatory acceptability [70].

Moreover, comparative analysis of the four composites has shown that Ag+PLA and Cu+PLA exhibited a slightly higher initial action of antimicrobials, probably because of higher ion release rates, whereas Ni+PLA and CuO+PLA achieved the same bactericidal levels at prolonged exposure [6,92]. This is consistent with previous accounts on the use of ions as antimicrobial agents, with silver and copper ions having a quicker effect on bacterial membranes, and nickel and copper oxide having a slower, ROS-mediated route [89,90,91]. Significantly, these findings indicate that material composition can be tailored for specific biomedical applications: silver- and copper-based composites for rapid disinfection of high-contact hospital surfaces, and nickel- and copper oxide–based composites for longer-lasting antimicrobial efficacy in coatings. In such differentiation, the prospects of 3D-printed materials can be highlighted as scalable alternatives to injection-moulded composites because of their high antimicrobial efficacy and economical usability in industrial settings [93]. Although the test of SEM and EDS indicated that CuO–PLA had the coarsest morphology and best incorporated the largest number of metallic species onto its surface, antimicrobial performance revealed that Ag–PLA and Cu-PLA composites possessed quicker initial bactericidal action [93]. This apparent contradiction between surface metal concentration and antimicrobial kinetics is common in nanoparticle/polymer systems and could be attributed to variations in ion-release behaviour, but not to surface abundance alone. Ag nanoparticles liberate Ag ions quickly upon being hydrated to create an immediate membrane-disruptive effect, and CuO nanoparticles tend to depend more on slower ROS-mediated processes and slower release of Cu^2+^. Consequently, despite the fact that CuO-PLA has a higher surface metal content, Ag-PLA has the capability to attain faster initial bactericidal activity. Similar results have been noted in the past, with the rate of antimicrobial governed largely by kinetics of ion-release, redox potential, and nanoparticle solubility, rather than the overall surface loading [94,95,96]. Thus, our findings are consistent with the available literature that Ag-based composites are frequently able to exhibit early-on antimicrobial action, whereas CuO-based systems are sufficiently robust but more time-dependent [94,95,96,97].

This seeming contradiction is because surface morphology is not the sole determinant of antimicrobial efficacy, but the actual processes of metal ion release and reactive oxygen species (ROS) production are determinants. Silver and copper nanoparticles are reported to release ions easily, which causes immediate destabilisation of the bacterial membrane. Nickel and copper oxide are reported to follow slower ROS-mediated mechanisms that may be observed over a long incubation period [89,90,91,93]. Thus, although SEM established the scope of embedded particles and the surface distribution, antimicrobial dynamics are more likely to be attributed to the synergistic dynamics of ion release of each system. The methodology thus enhances the existing body of knowledge beyond the validation of the antimicrobial activity of nanoparticle–PLA make-ups, to show their functionality when injection-moulded, a process of production that is directly pertinent to mass production. The comparative evaluation of several types of nanoparticles against clinically relevant Gram-positive and Gram-negative pathogens on a systematic basis under the same conditions further offers distinct knowledge towards specific health care requirements, in any case, in the formulation of composites [6,92,93,97]. While PLA-based composites are generally considered environmentally preferable, offering a significantly lower carbon footprint compared to conventional fossil plastics due to their renewable sourcing, the full environmental benefits depend on responsible sourcing and efficient lifecycle management. The fact that nanoparticle, especially silver, biosynthesis can be energy-intensive and add further greenhouse gas emissions into the equation means that it should be explored for a sustainability lifecycle assessment, if both biopolymer and nanoparticle stages are included, in the future, to ensure a net positive outcome [92,93,97,98,99].

Although the antimicrobial behaviour of Ni-PLA, Cu-PLA, Ag-PLA, and CuO PLA composites has been substantially demonstrated in this work, their use in biomedical research also differs according to biocompatibility, as known cytotoxic and sensitising behaviours have been recorded regarding some of the metals, such as nickel [100].

Even though cytotoxicity assays (fibroblast viability, keratinocyte response, or macrophage activation) were not the focus of the current research, they are a critical subsequent step prior to any biological or clinically relevant translation. It has been demonstrated before that nanoparticle–PLA matrices can regulate the release of ions and minimise direct cell exposure to metal nanoparticles, but material verification should be performed to ensure safe use. Systematic in vitro cytotoxicity testing with human fibroblasts and epithelial cell lines will thus be included in future work, alongside hemocompatibility testing to further understand the biomedical applicability of these injection-moulded composites [100,101,102,103].

## 6. Conclusions

In this study, the antimicrobial efficacy of the metal nanoparticles–PLA polymeric composites (Ag+PLA, Ni+PLA, Cu+PLA, CuO+PLA) was studied against a wide range of Gram-positive and Gram-negative bacterial strains commonly implicated in hospital-acquired infections (HAIs), like *Enterococcus faecalis*, *Staphylococcus aureus*, *Klebsiella pneumoniae*, *Salmonella Poona*, *Pseudomonas aeruginosa*, and *Escherichia coli*. Composites were developed with the help of injection moulding by integrating metallic nanoparticles like silver (Ag), nickel (Ni), copper (Cu), and copper oxide (CuO) in a specific ratio by weight into the PLA. The results demonstrated that all four developed polymeric composites were highly efficient against all the tested strains, with a reduction rate of nearly 98 percent in just one hour, except for Ni+PLA and Ag+PLA, which had slightly lower efficacy (97%) against Enterococcus faecalis and Pseudomonas aeruginosa. Furthermore, it was observed that all four composite materials maintained their antimicrobial effectiveness over the 24 h. The resulting metal–PLA polymeric composites exhibited good antimicrobial effects against Gram-positive and Gram-negative bacteria, wherein the bacterial viability was reduced to over 98 percent in less than an hour. The unique surface morphologies and ion release profiles of the composites are some attributes to their effectiveness and the possibility of their application in biomedical devices in the prevention of hospital-acquired infections. The study of characterisation revealed that CuO+PLA demonstrated the best surface incorporation, Ni+PLA the best thermal stability, and Ag+PLA the best crystallinity related to material properties and antimicrobial performance, respectively. All in all, CuO+PLA is the best candidate to use in antimicrobial implantation in bone tissue engineering because of its excellent surface activity and stable bactericidal action. Future studies must concentrate on the optimisation of nanoparticle delivery and thorough biocompatibility evaluation to ensure safe clinical use. The comparative study also reveals that the developed metal–PLA polymeric composites exhibit promising antimicrobial properties against a wide range of pathogens, with potential applications in manufacturing medical devices and surfaces with intrinsic antimicrobial properties. These materials would be extremely beneficial in the clinical realm, as hospital-acquired pathogens exist on surfaces for extended periods of time and lead to device-related infections. Future work should optimise nanoparticle concentrations and evaluate their long-term biocompatibility and safety profiles, while exploring the potential integration of these materials into a broader range of biomedical devices and hospital surfaces to realise their full clinical potential. Overall, this research shows that injection-moulded metal–PLA composites not only retain good antimicrobial properties but also offer an avenue of feasible production of medical devices and hospital equipment with in-built antimicrobial abilities. In contrast to 3D printing, injection moulding guarantees better reproducibility and scalability, as well as surface finish, which is essential in a medical setting. The combination of cytotoxicity tests, mechanical durability tests, and cost–benefit analysis must be involved in future research to speed up clinical translation. With high efficacy against clinically relevant Gram-positive and Gram-negative pathogens and scalable fabrication, these composites have the potential to reduce the burden of hospital-acquired infections and enhance patient safety in the global context.

## Figures and Tables

**Table 1:** Different nanoparticles with their properties.

Nanoparticle	Size (nm)	Surface Area (m^2^/g)	Density (g/cm^3^)	Purity	Structure and Morphology
**Copper**	80–240	4.6	8.9	99.95%	Crystal structure (Face-Centred Cubic (FCC)) reddish brown, with spherical shape and cubic crystal structure
**Copper Oxide**	15–45	15	6.5	99.995%	Crystal structure (monoclinic tenorite), dark brown, spherical in shape
**Silver**	28–48	15–18	10.6	99.95%	Crystal structure (Face-Centred Cubic (FCC)), black, spherical in shape, with a cubic crystal structure
**Nickel**	65	10–16	8.9	99.95%	Crystal structure (Face-Centred Cubic (FCC)), spherical and black in colour

**Table 2:** EDS quantitative analysis of the metal–PLA composites.

Composite	C (Mass%)	O (Mass%)	Metal (Mass%)	Metal (Atom%)	Metal Peakak Detected
**Ni+PLA**	52.50	46.67	0.83	0.19	Ni Kα (weak)
**Ag+PLA**	51.79	48.16	0.05	0.01	Ag Lα (trace)
**Cu+PLA**	51.11	48.78	0.11	0.02	Cu Kα (faint)
**CuO+PLA**	54.54	43.50	1.97	0.42	Cu Kα (strong)

**Table 3:** Presents the total count of bacteria for the control sheet at various time intervals for different bacterial species.

Sample	Challenged Bacteria	Inoculum	Recovered Bacterial Count					
			5 min	10 min	20 min	1 h	8 h	24 h
**Control Sheet**	***Staphylococcus aureus***	4500	4200	3850	3600	3110	2900	2650
	***Enterococcus faecalis***	3300	3100	2860	2550	2300	2150	1880
	***Salmonella Poona***	3500	3300	3050	2800	2250	2350	2100
	***E. coli***	4500	4200	3900	2650	2400	2150	2750
	***Klebsiella pneumoniae***	3700	3400	3150	2950	2650	2400	2100
	***Pseudomonas aeruginosa***	3400	3200	3000	2700	2550	2300	2000

**Table 4:** Presents the total count of bacteria for the Ni+PLA sheet at various time intervals for different bacterial species.

Sample	Challenged Bacteria	Inoculum	Recovered Bacterial Count					
			5 min	10 min	20 min	1 h	8 h	24 h
**Ni+PLA**	***Staphylococcus aureus***	4500	2800	920	410	55	1	1
	***Enterococcus faecalis***	3300	1800	890	390	80	1	1
	***Salmonella Poona***	3500	2000	950	350	60	1	1
	***E. coli***	4500	2500	1100	460	50	1	1
	***Klebsiella pneumoniae***	3700	2200	1020	370	40	1	1
	***Pseudomonas aeruginosa***	3400	2000	990	310	75	1	1

**Table 5:** Presents the total count of bacteria for the Ag+PLA sheet at various time intervals for different bacterial species.

Sample	Challenged Bacteria	Inoculum	Recovered Bacterial Count					
			5 min	10 min	20 min	1 h	8 h	24 h
**Ag+PLA**	***Staphylococcus aureus***	4500	2600	950	290	43	1	1
	***Enterococcus faecalis***	3300	1700	860	420	75	1	1
	***Salmonella Poona***	3500	1900	1000	370	65	1	1
	***E. coli***	4500	2400	1150	430	36	1	1
	***Klebsiella pneumoniae***	3700	2100	1090	340	48	1	1
	***Pseudomonas aeruginosa***	3400	1900	940	290	70	1	1

**Table 6:** Shows the total count of bacteria for the Cu+PLA sheet at different intervals of time for different bacteria.

Sample	Challenged Bacteria	Inoculum	Recovered Bacterial Count					
			5 min	10 min	20 min	1 h	8 h	24 h
**Cu+PLA**	***Staphylococcus aureus***	4500	2900	1010	415	35	1	1
	***Enterococcus faecalis***	3300	1900	880	500	56	1	1
	***Salmonella Poona***	3500	2200	910	390	45	1	1
	***E. coli***	4500	2700	1020	420	41	1	1
	***Klebsiella pneumoniae***	3700	2250	900	350	32	1	1
	***Pseudomonas aeruginosa***	3400	2100	850	305	66	1	1

**Table 7:** Shows the total count of bacteria for the CuO+PLA sheet at different intervals of time for different bacteria.

Sample	Challenged Bacteria	Inoculum	Recovered Bacterial Count					
			5 min	10 min	20 min	1 h	8 h	24 h
**CuO+PLA**	***Staphylococcus aureus***	4500	2650	1300	520	49	1	1
	***Enterococcus faecalis***	3300	1850	1060	610	56	1	1
	***Salmonella Poona***	3500	2150	990	465	73	1	1
	***E. coli***	4500	2560	1250	535	65	1	1
	***Klebsiella pneumoniae***	3700	2110	1100	390	38	1	1
	***Pseudomonas aeruginosa***	3400	1950	970	420	67	1	1

## Data Availability

The authors confirm that the data supporting the findings of this study are available within the article.
